# Social, forensic, and clinical correlates in female observandi referred for non-violent crimes

**DOI:** 10.4102/sajpsychiatry.v30i0.2209

**Published:** 2024-03-28

**Authors:** Muthumuni Nemavhola, Tando A.S Melapi, Danie Hoffman, Ora Gerber-Schutte

**Affiliations:** 1 Department of Psychiatry, Faculty of Health Sciences, University of the Witwatersrand, Johannesburg, South Africa

**Keywords:** female observandi, non-violent crimes, forensic, social, clinical, correlates

## Abstract

**Background:**

Globally, crime is highly masculinised and research into female criminality is scarce. In South Africa, no research specifically investigating the characteristics of female observandi referred for non-violent crimes has been published.

**Aims:**

The study aimed to describe the socio-demographic, clinical, and forensic correlates in women referred to Sterkfontein Hospital for forensic psychiatric observation following a non-violent criminal charge between 2010 and 2019. It also sought to establish the relationship between the correlates and fitness to stand trial and criminal responsibility, as well as the relationship between the socio-demographic characteristics and the different non-violent criminal charges.

**Setting:**

Sterkfontein Hospital.

**Methods:**

A retrospective record review of all cases referred to Sterkfontein Hospital for a single observation over 10 years was conducted.

**Results:**

Sixty-five cases were included in the study. Most observandi referred for non-violent crimes were found to be single (84.6%), unemployed (67.7%), reported abuse (55.4%), and had a high prevalence of mental illness (90.8%). Non-adherence to treatment was identified in 59.1% and substance use was identified in 72.2% of the study sample. The most common charge was theft (64.6%). The majority of the sample was found fit and responsible (57%). Bipolar (21%) and Primary Psychotic Disorders (35.7%) were associated with statistically significant outcomes of trial incompetence (*p* = 0.005) and lack of responsibility (*p* = 0.028).

**Conclusion:**

It is recommended that prospective studies are conducted which include comparisons with male counterparts and females referred for violent crimes.

**Contribution:**

The study identified correlates that should be included in the standard of care in forensic assessments of female observandi.

## Introduction

Research into the understanding of female criminality is scarce both globally and locally.^1^ In South Africa, criminality is highly masculinised with women accounting for only a small percentage of offenders.^2^ The South African Department of Correctional Services reported that the number of female detainees in March 2019 was only 4316 (3%) versus 158 559 (97%) males.^3^ International and local studies have shown that women exhibit fundamentally less violent criminal behaviour than their male counterparts.^1,2,4,5^ The Uniform Crime Reports of the Federal Bureau of Investigations found that non-violent crimes were more prevalent in women. For example, non-aggravated assault, drug offences, and driving under the influence of alcohol accounted for 45% of women’s arrests.^4^

A German-Greek self-report study found a distinct gender gap for violent and property offences.^6^ The latter research study also found that women were twice as likely to commit shoplifting than males.^6^ A Turkish study found that both men and women were involved to a greater extent in minor property crimes than in violent crimes. The last-mentioned study also found that 80% of the female offenders had witnessed or experienced violence by their parents or intimate partners before their arrest.^7^ Even though most women are charged with non-violent crimes, very few research studies focused on women charged with non-violent crimes and referred for psychiatric evaluation.^1^

In the South African context, psychiatrists in criminal forensic psychiatry are tasked with, among others: determining whether a person accused of a crime may have a mental illness that renders him or her unable to understand court proceedings (fitness to stand trial) and whether such a mental illness may have caused the person, at the time of the offence, to be incapable of appreciating the wrongfulness of the act and/or act in accordance with such an appreciation (criminal responsibility).^8,9^ During a 30-day observation period, the accused is assessed for fitness to stand trial in terms of section 77 and criminal responsibility under section 78 of the Act.^9^ In a case where the accused is charged with murder, culpable homicide, rape, or another crime involving serious violence, the observandus is referred for forensic assessment by a panel of psychiatrists.^9^ Usually, observandi referred for non-violent crimes are referred for a single observation either by the medical superintendent of a psychiatric hospital or a psychiatrist appointed by the medical superintendent.^9^ During the 30-day observation period the observandi undergo physical examinations, psychiatric evaluations, social interventions, and evaluations by other members of the multi-disciplinary mental health team including psychologists, occupational therapists, and social workers. Those found not fit to stand trial and/or not criminally responsible are referred by the courts for admission as if they are involuntary mental health care users under section 37 of the *Mental Health Care Act* of 2002.^9^

Previous studies conducted on female observandi referred for forensic assessment under the *South African Criminal Procedure Act* (CPA) of 1977 mainly focused on women referred for violent crimes.^1,10,11,12,13^ Several local and international research studies have found that women with mental illnesses are at higher risk of being charged with both violent and non-violent crimes.^1,10,12,13,14^ Furthermore, a research study conducted in a Durban prison found that over 50% of female prisoners had a psychiatric disorder or disorders.^15^ Similarly, in a pilot of legally referred female shoplifters, Silverman and Brener found that there was a clinically recognisable pattern of psychiatric symptoms in their 10-year UK-based cohort.^14^ Naidoo et al.^16^ found a high prevalence (90.4%) of mental disorders in their sample of female prisoners in Durban, South Africa. It is evident from the literature review discussed earlier that there is a paucity of research into female observandi referred for non-violent crimes.^16^

## Aim and objectives

The study aimed to describe the socio-demographic, clinical, and forensic correlates in females accused of non-violent crimes and referred for forensic observation to a specialised forensic psychiatric hospital in Gauteng, South Africa under the CPA.^9^ The objectives of the study were:

- To describe the socio-demographic characteristics (age, marital status, employment status, and domestic violence history) of the study population.
- To describe the clinical characteristics (psychiatric diagnosis, substance use, and treatment adherence) of the study population.
- To determine if any associations exist between the clinical characteristics and fitness to stand trial and criminal responsibility.
- To determine if any associations exist between the socio-demographic characteristics and the different types of non-violent criminal charges.

## Research methods and design

### Study design and setting

A retrospective record review of all women referred by courts to Sterkfontein Hospitalfor a non-violent criminal charge over a 10-year period (2010–2019), was conducted. Sterkfontein Hospitalis a specialised forensic and general psychiatry hospital which serves courts in the South of Gauteng and the Northwest province with forensic assessments.

### Study population

All women above the age of 18 charged with a non-violent offence and who were admitted to Sterkfontein Hospital for observation in terms of section 79 of the CPA from 01 January 2010 until 31 December 2019, were included in the study. For this study, all female observandi referred for observation by a panel (2 or more psychiatrists) were defined as being charged with a violent crime, and those referred as a single observation were defined as having a non-violent charge. Any female observandi under the age of 18 were excluded from the study. Some of the female observandi referred for non-violent crimes who were ordered to undergo observation by a panel because of multiple fraud or theft charges or charges involving large amounts of money were also excluded from the study as only those referred for a single observation were regarded as being charged with a non-violent charge.

### Data collection

*Criminal Procedure Act* report files located in the forensic unit of the hospital were accessed first as they were filed according to the year that the accused persons were referred for observation. The front page of each file had an index page containing the gender and charge of the accused. The number on the index page was used to find the psychiatric report to determine whether the accused was referred for a single or panel observation. Using the file number from the psychiatric report, the clinical file of the female accused referred for a non-violent criminal charge was then drawn from the hospital registry for data collection. The data were captured using a data-collection sheet that included all the studied variables. One hundred and eleven women referred for forensic observation during the period under review were eligible for inclusion in the study. Of the 111 eligible observandi only 65 medical record files were found in the hospital’s registry, and these were included in the final data collection.

### Ethical considerations

The protocol was approved by the University of the Witwatersrand Johannesburg’s Human Research Ethics Committee (clearance certificate No: M220927). The study had also been registered on the National Health Research Database (Ref no: GP 202111_036). Permission to collect data from Sterkfontein Hospital was obtained from the hospital’s research committee and the head of the health establishment. All the data were collected and captured by the principal investigator who ensured anonymity, security of the data, confidentiality, and compliance with the *Protection of Personal Information (POPI) Act* for research by not capturing any unique identifiers (personal information) onto the data-collection sheet.

### Data analysis

All statistical analyses were conducted using Datatab (https://datatab.net/). Continuous variables such as age were analysed using means and standard deviations. Categorical variables such as criminal charge, marital status, and employment status were analysed using frequencies (percentages). The degree of association between categorical variables was assessed using the Pearson chi-square test as it is a non-parametric test that does not require a null hypothesis or any assumptions to be made. The significance level was set at a p-value of less than 0.05.

## Results

### Socio-demographic correlates

The mean age of the female accused referred for non-violent crimes was 39 years (standard deviation [s.d.]: 11.4) with the highest proportion in the 30–49 age group (52%), followed by the 18–29 age group (26%) and those aged 50 and above accounted for 22% of the sample. The minimum age was 19 years while the maximum age was 63 years. More than two-thirds (84.6%) of the sample were single, divorced, or widowed at the time of observation and 15.4% were married, cohabitating, or in a relationship. About 55% of the sample had a self-reported history of domestic violence or abuse whilst only 9% denied any prior history of domestic violence or abuse. A history of domestic violence or abuse was not documented in 35.4% of the sample. Regarding socio-economic status, 67.7% were unemployed whilst 18.5% were employed, and 13.8% received a grant. The socio-demographic correlates are summarised in Table 1.

**TABLE 1:** Socio-demographic correlates.

Categories	Frequency	Percentage
**Age category**		
18–29 years	17	26.2
30–49 years	34	52.3
50 years and older	-	21.5
**Marital status**		
Married, in a relationship or cohabitating	10	15.4
Single, divorced, or widowed	55	84.6
**Employment status**		
Unemployed	44	67.7
Employed	12	18.5
On a grant	9	13.8
**Domestic violence history**		
History of domestic violence	55	55.4
No domestic violence history	6	9.2
Unknown	23	35.4

### Clinical correlates

The ICD-10 classification of mental and behavioural disorders was used to categorise diagnoses in the study (Table 2). The three commonest diagnostic categories were affective disorders (bipolar and depressive disorders), organic mental disorders (psychiatric disorders because of another medical condition), and primary psychotic disorders (schizophrenia, schizoaffective, and delusional disorders). A majority (90.8%) of female observandi referred in the period under review were diagnosed with a psychiatric condition at observation. The commonest diagnostic category was affective (mostly bipolar and major depressive) disorders (33.8%), followed by organic mental disorders (16.9%), and primary psychotic disorders (15.4%).

**TABLE 2:** Diagnoses at observation.

Diagnosis (ICD 10 codes)	Frequency	Percentage
Mood (affective) disorders (F31–F39)	22	33.8
Organic, including symptomatic, mental disorders (F0–F9)	11	16.9
Schizophrenia, schizotypal, and delusional disorders (F20–F25)	10	15.4
Disorders of personality and behaviour in adult persons (F60–F69)	10	15.4
Mental and behavioural disorders because of the use of psychoactive substances (F10–F19)	8	12.3
No mental illness	6	9.2
Behavioural syndromes associated with physiological disturbances and physical factors (F50–F59)	3	4.6
Mental retardation (F70–F79)	2	3.1
Neurotic, stress-related, and somatoform disorders (F40–F49)	1	1.5

Seventy-two per cent of the sample reported substance use at the time of the offence. The most used substance was alcohol, followed by cannabis, and stimulants (see Table 3). Regarding adherence to psychotropic medication, a total of 49 out of the 65 (75.38%) female observandi were on treatment at some point prior to referral for observation. Of those who were on treatment, more than 60% were non-adherent whilst only 24.5% were adherent. Adherence status was not documented in 14.3% of the sample.

**TABLE 3:** Substance use.

Substance	Frequency (*N* = 97)	Percentage
No substance use	27	27.8
Alcohol	20	20.6
Cannabis	16	16.5
Stimulants	14	14.4
Other substances	12	12.4
Opioids	8	8.2

### Forensic correlates

The charges were grouped into three categories: property crimes (theft, malicious injury to property [MITP], fraud, and trespassing), crimes against the person (assault, contravention of protection order, and culpable homicide), and crimes against the state (possession of drugs and cruelty to animals). A high number (83.1%) of the females accused were charged with property-related crimes followed by crimes against the person (9.2%). Crimes against the state were in the minority (7.7%). The two commonest individual charges (Table 4) were theft (64.6%) and MITP (12.3%). As illustrated in Table 5, the majority of the observandi were found criminally responsible (58%), whilst 42% were found not criminally responsible. The majority of the sample was found fit to stand trial (77%). Only 22% were found both not fit to stand trial and not criminally responsible.

**TABLE 4:** Individual charges.

Charge	Frequency	Percentage
Theft	42	64.6
MITP	8	12.3
Assault	2	3.1
Possession of drugs	3	4.6
Fraud	3	4.6
Contravention of protection order	3	4.6
Trespassing	2	3.1
Using of drugs	1	1.5
Cruelty to animals	1	1.5

MITP, malicious injury to property.

**TABLE 5:** Fitness to stand trial and criminal responsibility.

Fitness	Responsible		Not responsible		Total	
	*N*	%	*N*	%	*N*	%
Fit	37	57	13	20	50	77
Not fit	1	2	14	22	15	23
**Total**	**38**	**58**	**27**	**42**	**65**	**100**

### Forensic and clinical correlates relationship

#### Fitness to stand trial, criminal responsibility and psychiatric diagnosis

More than a third (35.71%) of those found not fit and not responsible were diagnosed with a primary psychotic disorder whilst 21% were diagnosed with bipolar disorder. A Chi-squared (χ^2^) test was conducted between bipolar disorder and responsibility, at least one of the expected cell frequencies was less than 5. Therefore, the assumptions for the χ^2^ test were not met. There was a statistically significant relationship between bipolar and being found not criminally responsible, χ^2^ (1) = 7.94, *p* = 0.005, Cramér’s *V* = 0.35. A further logistic regression analysis was conducted to examine the influence of bipolar disorder on fitness to stand trial. This logistic regression analysis showed that the model as a whole was not significant (χ^2^(1) = 1.44, *p* = 0.23, *n* = 65) so the likelihood that observandi diagnosed with bipolar would be found not fit to stand trial was not statistically significant.

Another χ^2^ test was conducted between psychotic disorder and responsibility. At least one of the expected cell frequencies was less than 5. There was a statistically significant relationship between psychotic disorder and a lack of responsibility (χ^2^ (1) = 7.2, *p* = 0.007, Cramér’s *V* = 0.33). There was a statistically significant relationship between the diagnosis of a primary psychotic disorder and being found not fit for trial (χ^2^(1) = 4.21, *p* = 0.04, *n* = 65).

All participants diagnosed with a personality disorder (15.4%) were found to be both fit to stand trial (χ^2^(6) = 10.61, *p* = 0.101, *n* = 65) and criminally responsible (χ^2^(6) = 4.41, *p* = 0.621, *n* = 65).

#### Different types of crimes and psychiatric diagnoses

Among those charged with property-related crimes, the top three diagnoses were affective disorder, personality disorders, and psychotic disorders (33.3%, 18.5%, and 16.67% respectively). Fifty per cent of the sample referred for crimes against the person were diagnosed with organic psychosis whilst 30% were diagnosed with an affective disorder. Twenty per cent were not diagnosed with any mental illness. Forty per cent of those women referred for crimes against the state were diagnosed with a substance-related disorder, 40% an affective disorder, and 20% with a primary psychotic disorder. Forty-four (67.69%) of those who used substances were found to be criminally responsible.

#### Substance use and criminal responsibility

Logistic regression analysis was conducted to examine the influence of substance use on criminal responsibility. The logistic regression analysis showed a statistically significant relationship between substance use and being found criminally responsible (χ^2^(1) = 7.19, *p* = 0.007, *n* = 65).

### Socio-demographic and forensic correlates relationship

#### Age and type of crime

Table 6 shows the top five charges and the mean age for each charge. Theft had the highest mean (SD) age at 42 (SD: 10.33) and possession of drugs had the lowest at 24 (SD: 4.58). Logistic regression analysis which was conducted to examine the influence of age on charge category showed with statistical significance that an increase in age is associated with an increase in the probability that the female observandus was charged with a crime against the person (χ^2^(5) = 48.49, *p* < 0.001, *n* = 65). For crimes against the state, an increase in age was associated with a decrease in the probability that the observandi were charged with a crime against the state (χ^2^(1) = 7.51, *p* = 0.006, *n* = 65). Regarding crimes against the person, there was a statistically significant probability that the observandi were charged with a property-related crime with increasing age (χ^2^(1) = 8.16, *p* = 0.004, *n* = 65).

**TABLE 6:** Top five charges with mean ages.

Charge	Frequency	Mean	s.d.
Theft	42	40.14	10.33
MITP	7	39.57	11
Assault	4	29	5.29
Possession of drugs	3	24	4.58
Contravention of protection order	3	37.67	17.24
Fraud	2	38	5.66

s.d., standard deviation; MITP, malicious injury to property.

#### Employment status and type of crime

Logistic regression analysis was conducted to examine the influence of employment status on the charge category. The analysis showed that the relationship between employment status and being charged with a property crime (χ^2^(2) = 5.64, *p* = 0.06, *n* = 65), crimes against the state (χ^2^(2) = 4.97, *p* = 0.083, *n* = 65), or crime against the person (χ^2^(2) = 3.84, *p* = 0.147, *n* = 65) was not statistically significant.

#### Marital status and type of crime

Further analysis was conducted to examine the influence of marital status on the charge category. There was a statistically significant relationship between being unmarried (single, divorced, or widowed) (χ^2^(1) = 6.92, *p* = 0.009, *n* = 65) and being charged with a property-related crime. Conversely, the relationships between being married and crimes against the state (χ^2^(1) = 2.35, *p* = 0.126, *n* = 65), as well as crimes against the person (χ^2^(1) = 3.19, *p* = 0.074, *n* = 65), were not statistically significant.

## Discussion

### Socio-demographic characteristics

Calitz found that the mean age of accused males and females was 30 years,^12^ whilst Ndala found that the mean age for males and females ranged between the ages of 21 and 30.^11^ Both Du Plessis et al.^13^ and Nagdee et al.^1^ found that the highest percentage of observandi in their respective studies was in the age group of 20–29 years. Conversely in this study, the mean age was higher at 39 years and the age group with the highest proportion fell within the 30–49 years range. The difference between previous literature and the finding of the current study may be attributable to the fact that all the above-mentioned studies reviewed participants who had been referred by the courts for both violent and non-violent criminal charges.^1,3,11,12^ In comparison to previous research, findings from this study suggest that the female observandus referred specifically for non-violent crimes is older than the woman who is referred for violent criminal charges.^1,11,12^

Similar to other studies investigating female offenders referred for psycho-legal assessment, the majority (84.6%) of the women in this study were unmarried.^1,12,17^ Comparable to the Nagdee et al study,^1^ (78%), the unemployment rate in the study was high (67.7%) which could be an explanation for the high incidence (64.6%) of the charge of theft in the sample. More than half of the sample (55.4%) reported a history of domestic violence by parents or a partner and this was in keeping with the previous studies that reported a high incidence of reported abuse.^1,7^

### Clinical characteristics

Bipolar disorder, schizophrenia, and psychiatric disorders because of another medical condition were the commonest diagnoses. Previous research studies found that women with mental illnesses are at higher risk of being charged with both violent and non-violent crimes.^1,10,12,13,14^ The findings of this study indicated that most women charged with non-violent crimes had a mental illness. In a pilot of legally referred female shoplifters, Silverman and Brener^14^ found that there was a clinically recognisable pattern of psychiatric symptoms in their 10-year cohort. The symptoms included marital disharmony, depressed mood, and anxiety amongst others. Locally, Ndala^11^ and Du Plessis^13^ found that schizophrenia, intellectual disability, and substance-induced psychotic disorders were the commonest psychiatric diagnoses in their respective observandi study populations. Similarly, Calitz et al.^12^ found that schizophrenia was the most common diagnosis followed by bipolar disorder, epilepsy, substance-induced psychotic disorder, and intellectual disability.

A significant proportion of the sample in the current study (90.8%) had a diagnosable mental illness: this was slightly higher than what was seen in other studies and points to the high threshold to refer accused who are charged with non-violent crimes. The detrimental consequence of this was classically observed in the two cases of women who were diagnosed with kleptomania at observation, who were each only referred for assessment after multiple convictions of theft. Furthermore, Nagdee et al.^1^ found that amongst those women who were diagnosed with a mental illness, there was a high incidence of substance use, mood and psychotic spectrum disorders, and a lower incidence of personality disorders. Similar to Nagdee et al.,^1^ this study found a combined incidence of bipolar disorder (32.3%) and primary psychotic disorders (15.4%) of 47.7%.

Regarding adherence to psychotropic medication, it was found that over 60% of the sample in this study were non-adherent. This was also evident in Ndala’s^11^ finding that non-adherence to treatment leads to offending behaviour that exposes observandi to contact with the criminal justice system. Similar to other international and local studies,^1,11,12,14^ the incidence of substance use was high in the sample with almost 60% of the women reporting substance use at the time of the crime.

### Forensic correlates

As observed in other research most (83%) women in this study were charged with economic and property-related crimes.^1,14^ Despite the high number of diagnosed psychiatric conditions in the sample more than half of the sample was found to be both fit to stand trial and criminally responsible (57%). This observation is universal to all the studies reviewed and points to the need to carefully select candidates for observation in a resource-constrained context such as South Africa.^1,11,12,13^ The reasons for the high fitness to stand trial rates despite a high prevalence of mental illness may be attributable to the mental illness being treated before the commencement of observation (because of long waiting periods between the offence and observation). There was a statistically significant relationship between a diagnosis of bipolar disorder or a primary psychotic disorder and being found both unfit to stand trial and not criminally responsible. On the other hand, the relationship between being diagnosed with a personality disorder and being found criminally responsible was not statistically significant.

## Conclusion

From the findings of the study, female observandi referred for non-violent crimes can be described as single and unemployed women, with a history of reported abuse, having a high prevalence of mental illness and substance use, who tend to be charged with theft or shoplifting. There were statistically significant relationships between bipolar and psychotic disorders and being found not criminally responsible. Those diagnosed with substance use disorders and personality disorders were likely to be found criminally responsible. Those diagnosed with psychotic disorders were also found to be not fit to stand trial. There was a likelihood of being charged with a property-related crime or a crime against the person with increasing age whilst younger women were likely to be charged with a crime against the state.

This is a pioneer study that specifically describes the correlates of female observandi referred for non-violent crimes in South Africa. Although it was a small single-site investigation, it highlighted the characteristics of the woman referred for non-violent crimes that distinguish her from the woman or man referred for violent crimes.

The identified correlates should be included in the standard of care in forensic assessments of female observandi. Clinicians in the general psychiatry setting should also be able to identify those women who are likely to commit non-violent crimes and ensure adherence to treatment and interventions to reduce substance use to prevent criminality. It is further recommended that prospective studies are conducted that include comparisons with male counterparts and females referred for violent crimes.

### Limitations

The retrospective nature of the study, however, limited the sample size and completeness of the information as some (45) of the files were not found and the recording of information varied between individual clinicians. For example, almost a third of the sample (35.4%) had an undocumented history of previous exposure to violence and abuse. Previous research has shown that exposure to violence predisposes to criminal behaviour,^2,10,18^ thus it was important to determine the abuse history of all the women in the study; however, the incompleteness of the data has underestimated the frequency of domestic violence in the sample.

There was no head-to-head comparison with the profiles of men referred for non-violent crimes. Recommendations are for future prospective research to be conducted in a multi-site study that includes both men and women who have been referred for non-violent crimes Future qualitative research could also provide greater insight into the factors that drive female observandi into committing non-violent crimes and their experiences of incarceration and psychiatric observation. The information gathered in this study is valuable in that it describes the characteristics that distinguish the female mental health care user likely to commit a non-violent crime and gives the clinician a unique opportunity to intervene and prevent the negative consequences of imprisonment.
